# Probing Saltern Brines with an Oxygen Electrode: What Can We Learn about the Community Metabolism in Hypersaline Systems?

**DOI:** 10.3390/life6020023

**Published:** 2016-06-08

**Authors:** Aharon Oren

**Affiliations:** Department of Plant and Environmental Sciences, The Alexander Silberman Institute of Life Sciences, The Hebrew University of Jerusalem, Edmond J. Safra Campus, 91904 Jerusalem, Israel; aharon.oren@mail.huji.ac.il; Tel.: +972-2-658-4951

**Keywords:** salterns, halophilic, hypersaline, oxygen, *Halobacteria*, *Haloquadratum*, *Salinibacter*, bacteriorhodopsin

## Abstract

We have explored the use of optical oxygen electrodes to study oxygenic photosynthesis and heterotrophic activities in crystallizer brines of the salterns in Eilat, Israel. Monitoring oxygen uptake rates in the dark enables the identification of organic substrates that are preferentially used by the community. Addition of glycerol (the osmotic solute synthesized by *Dunaliella*) or dihydroxyacetone (produced from glycerol by *Salinibacter*) enhanced respiration rates. Pyruvate, produced from glycerol or from some sugars by certain halophilic *Archaea* also stimulated community respiration. Fumarate had a sparing effect on respiration, possibly as many halophilic *Archaea* can use fumarate as a terminal electron acceptor in respiration. Calculating the photosynthetic activity of *Dunaliella* by monitoring oxygen concentration changes during light/dark incubations is not straightforward as light also affects respiration of some halophilic *Archaea* and *Bacteria* due to action of light-driven proton pumps. When illuminated, community respiration of brine samples in which oxygenic photosynthesis was inhibited by DCMU decreased by ~40%. This effect was interpreted as the result of competition between two energy yielding systems: the bacteriorhodopsin proton pump and the respiratory chain of the prokaryotes. These findings have important implications for the interpretation of other published data on photosynthetic and respiratory activities in hypersaline environments.

## 1. Introduction

**Figure 1:**
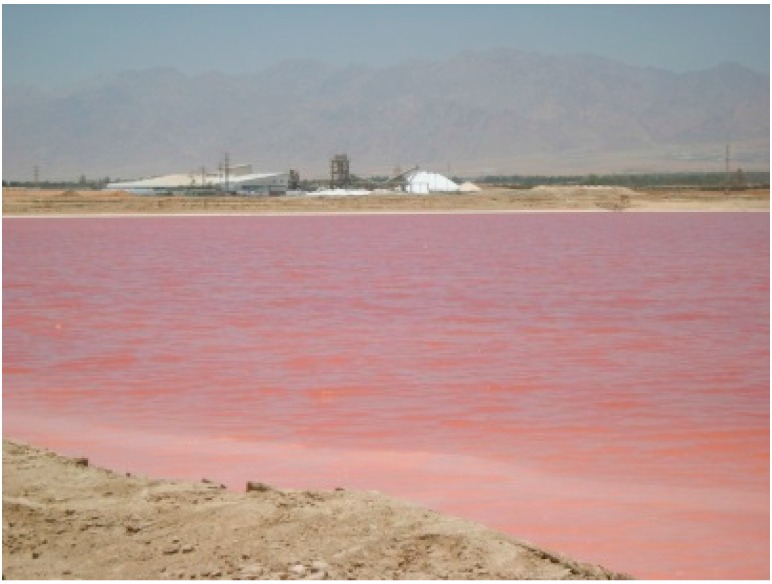
A crystallizer pond of Salt of the Earth, Ltd., Eilat, Israel.

Thanks to the recent advances in the methodology of metagenomics and other cultivation-independent molecular methods used in microbial ecology we now have a fairly complete picture, qualitative as well as quantitative, of the microorganisms that inhabit the crystallizer ponds of solar salterns worldwide. The NaCl-saturated brines of crystallizer ponds are typically inhabited by a single primary producer, the unicellular alga *Dunaliella salina* (*Chlorophyceae*) [1], and by 10^7^–10^8^ prokaryotes/mL [2,3,4]. Most of these belong to the archaeal domain: *Haloquadratum*, *Halorubrum*, and other members of the class *Halobacteria*, but also some members of the *Bacteria* can be found, notably of the genus *Salinibacter* (*Bacteroidetes*) [5,6,7]. Most organisms inhabiting the brines are pigmented pink, red, or orange due to carotenoid pigments: primarily β-carotene in *Dunaliella*, α-bacterioruberin and derivatives in the *Halobacteria*, and salinixanthin in *Salinibacter* [8]. As a result, the waters of saltern crystallizers are generally colored brightly red-pink (Figure 1). Retinal-containing membrane-bound proteins such as bacteriorhodopsin and similar proton pumps in the *Halobacteria* and xanthorhodopsin in *Salinibacter* may also contribute to the pigmentation.

Our understanding of the *in situ* activities of the different components of the biota of the crystallizer ponds lags behind our knowledge about the community composition. However, the high community densities present and the easy accessibility of most saltern pond systems make the salterns perfect objects for basic studies on microbial ecology at saturating salt concentrations. They are convenient model systems to explore basic questions about the primary productivity, the overall heterotrophic activity and the possible functions of different carbon sources that support the dense communities of *Archaea* (>85%–90%) and to lesser extent *Bacteria* present, and to elucidate the role of bacteriorhodopsin and other light-dependent proton pumps in the energy metabolism in the brines. However, thus far, the number of such studies, reviewed in the sections below, is surprisingly small.

I here summarize our recent attempts to obtain quantitative information about these processes in the saltern crystallizer ponds of Salt of the Earth Ltd., Eilat, Israel, based on measurements of changes in dissolved oxygen concentrations in the brine following different manipulations. Many of these experiments were performed by students in the framework of the annual course in marine microbiology for graduate students held in Eilat. The results show that much information can be gained about the basic processes that drive the biology of the saltern ponds by use of optical oxygen sensors (optodes) and simple experimental systems.

## 2. Dissolved Oxygen Concentrations in Crystallizer Brines

The solubility of oxygen and other gases in concentrated brines is greatly decreased compared to the values in freshwater or in seawater of the same temperature. In equilibrium with the atmosphere, NaCl-saturated brine of 260 g/kg salinity (~320 g/L dissolved salts) contains about 1.61 mg/L (50 μM) dissolved oxygen at 25 °C. At 35 °C, a temperature typically found in saltern brines during the summer season, the value is even lower, around 1.51 mg/L (47 μM). These values must be compared to 8.22 mg/L and 6.92 mg/L for freshwater and 6.98 and 5.95 mg/L for seawater at 25 °C and 35 °C, respectively [9,10].

The low solubility of oxygen in salt-saturated brines and the potentially high heterotrophic activity of the dense biota often result in near-anaerobic conditions in the crystallizer ponds, as the (probably little) oxygen produced by *Dunaliella* is rapidly taken up for respiration by the dense community of heterotrophic *Archaea* and *Bacteria*, especially at the high temperatures of such brines in tropical and subtropical areas [2,11]. Based on measurements by a chemical method (the Winkler titration in which molecular oxygen oxidizes Mn(II) to Mn(III) which in turn oxidizes iodide to iodine which is then titrated with thiosulfate), oxygen concentrations as low as 0.50 mg/L (16 μM) were measured in saltern crystallizer ponds in Spain [12]. A higher value (1.87 mg/L, 58 μM) was found in crystallizer brines of a Bulgarian salt works [13]. The lowest oxygen concentrations encountered in the literature for crystallizer pond waters are probably the values of 0.24 and 0.08 mg/L (7.5 and 2.5 μM) for Australian crystallizer brines with densities of 1.224 and 1.235 g/mL, respectively [14].

The low oxygen solubility in hypersaline brines it not necessarily a disadvantage for *in situ* activity studies. On the contrary, it can be advantageous, as small oxygen concentration changes can be sensitively recorded against the low background value. Even if the activity per cell in such salt-stressed systems may be low, the high community density enables the monitoring of changes in oxygen concentration within a time frame of a few hours.

## 3. Methods for Monitoring Oxygen Concentration Changes in Saltern Crystallizer Brines for the Estimation of Microbial Activities

There are very few records in the older literature of attempts to assess the rates of photosynthesis and/or respiration in saltern crystallizer ponds. Pedrós-Alió and coworkers incubated brines (250–280 g/L salt, 6–7 × 10^7^ prokaryotic cells/L) from the salterns at Bras-del-Port, Alicante, Spain in 150-mL BOD bottles in the dark for 4 h and measured the changes in oxygen concentration by the Winkler titration. Apparent respiration rates were below the detection limit of 0.3 μmol O_2_/L·h [15].

In 2008 we first used optical oxygen sensors (‘optodes’) to estimate microbial activities in the Eilat saltern crystallizer brines [16]. For these experiments the microbial community was first concentrated 24-fold by centrifugation to a final density of 7.9 × 10^8^ cells/mL to improve the detection limit and the sensitivity of the assays that lasted up to 30 min. Centrifugation was performed under relatively mild conditions (1500× *g*, 35 min, 20 °C). However, the possibility must be taken into account that this treatment may have damaged part of the cells, caused the loss of smaller cells, and/or had ruptured *Dunaliella* cells accompanied by the release of intracellular glycerol to the medium. The *Dunaliella* cells were lighter than the brine and did not enter the cell pellet. Samples of the resulting cell concentrate, diluted with 20% (by volume) of distilled water to increase uptake rates were placed in custom-built air-tight closed cuvettes provided with an optical oxygen sensor spot (SP-PSt3-YOP-PSUPD5) connected to a Fibox 3 device (PreSens GmbH, Regensburg, Germany). The resulting cell preparation (final cell density 6.3 × 10^8^/mL) gave a decrease of ~1% of oxygen saturation level in 10 minutes. The respiration rate was estimated at ~3 fmol/cell·h [16].

Parallel experiments were performed in which brine samples, whether or not enriched with different carbon sources (see below), were incubated in completely filled 50-mL BOD bottles and incubated in the dark at 30 °C. At time zero (O_2_ concentration ~20–27 μM) and after different incubation periods (3–4 time points up to 40–50 h) the residual oxygen concentration in the bottles was assayed in triplicate by Winkler titration [16].

**Figure 2:**
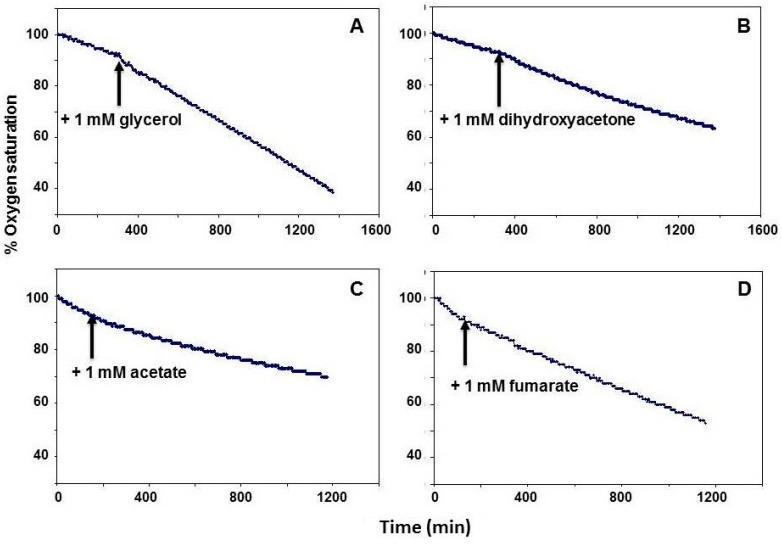
Changes in the respiration rate by the microbial community from a saltern crystallizer pond in Eilat following addition of glycerol (**A**); dihydroxyacetone (**B**); Na-acetate (**C**) or NaOH-neutralized fumaric acid (**D**). Portions of 630 mL NaCl-saturated brine from a crystallizer pond sampled in May 2011, and containing 2.9 × 10^7^ prokaryote cells/mL and 1200 *Dunaliella salina* cells/mL, were incubated in the dark in completely filled 630-mL Plexiglas chambers, each provided with a Yellow Springs Instrument optical oxygen electrode (Pro20 Lab/Field Dissolved Oxygen Meter) and a magnetic stirring bar, the temperature being controlled at 30–31 °C. The oxygen concentration was recorded every 5 min. At the time indicated by arrows, the carbon sources were added to a final concentration of 1 mM by injection of 0.63 mL of 1 M solutions of the respective compounds, and the effect of the substrate was estimated by the change in the oxygen uptake rate.

**Figure 3:**
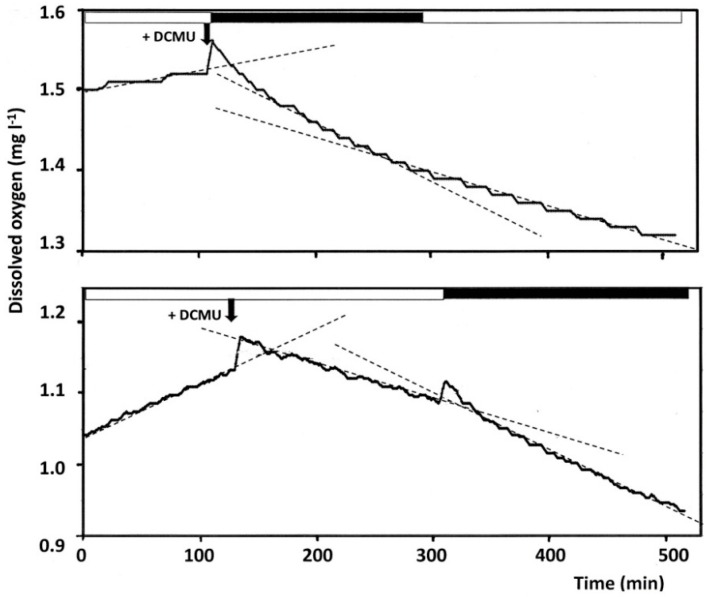
Kinetics of oxygen evolution and oxygen consumption in the light and in the dark by Eilat crystallizer brine, sampled in May 2015, at 35 °C in the presence and in the absence of DCMU. Chambers (630 mL) equipped with oxygen and temperature sensors and surrounded by a water jacket for temperature control were completely filled with brine from a crystallizer pond of the Eilat salterns. After temperature equilibration in the light (200–220 μmol quanta/m^2^·s,) for 70 min, changes in dissolved oxygen concentration were recorded. At the time indicated by the arrows, DCMU (5 μM) was added from a 5 mM solution in ethanol, and illumination was turned off and on as indicated by the white (light) and black (dark) bars at the upper part of each panel. The slopes from which the kinetics of net photosynthesis and respiration were calculated are indicated by the dashed lines, all based on data collected at a temperature of 35 ± 0.3 °C. The brine had a density of 1.202 g/mL at the *in situ* temperature of 35 °C, contained ~3.5 × 10^7^ prokaryotes/mL with >70% flat square cells, 2170 *Dunaliella* cells/mL, 0.8 μg/L chlorophyll *a*, 0.28 mg/L β-carotene, 0.098 mg/L bacterioruberin carotenoids, and ~3.6 nmol/L bacteriorhodopsin and other retinal proteins. From Extremophiles Vol. 20, 2016, p. 75, A. Oren *et al.* [18], with permission of Springer.

All later experiments described below were performed using commercial optical oxygen electrodes with data loggers (Yellow Springs Instrument optical oxygen electrode, Pro20 Lab/Field Dissolved Oxygen Meter, Yellow Springs, OH, USA), mounted in 630-mL Plexiglas temperature-controlled (30 or 35 °C) incubation chambers provided with magnetic stirring bars [17,18]. Sample temperature was continuously recorded by the temperature sensor included in the oxygen electrode setup. The oxygen optodes were calibrated by bubbling brine samples with nitrogen and with 100% oxygen, and the salinity setting was adjusted to that of the sample. All systems were left to equilibrate for at least one hour before measurements started. When indicated, systems were illuminated by two halogen lamps (full spectrum light, 500 W) at an incident light intensity of 200–220 μmol quanta/m^2^·s as measured using a Li-Cor LI-190 SA quantum sensor connected to a Li-Cor LI-1000 data logger. Figure 2 and Figure 3 provide examples of such experiments.

## 4. Effects of Selected Carbon Compounds on the Community Respiration in Saltern Crystallizer Ponds

The effects of the different substrates tested on the community respiration rates, using the three above-described experimental systems, can be summarized as follows [16,17]:

1. Significant stimulation of oxygen uptake was observed when brine samples were enriched with 1 mM of glycerol, dihydroxyacetone or pyruvate.
2. No or little stimulation was obtained following addition of 10 mg/L yeast extract.
3. No stimulation or even a slight inhibition of community respiration was found after addition of 1 mM Na-acetate or 1 mM succinate.
4. The oxygen uptake rate was significantly inhibited (up to 50%) following the addition of 1 mM fumarate.

Figure 2 shows representative examples of such substrate addition experiments in native brine samples in the 630-mL incubation chambers.

### 4.1. Glycerol

Glycerol is the osmotic solute synthesized by *Dunaliella*. Therefore it may be expected to be one of the main carbon compounds available to the heterotrophic communities in saltern crystallizer ponds [19]. Thus it was not surprising that the addition of 1 mM glycerol led to up to ~80% enhancement of respiration rates [16,17] (Figure 2A). Earlier measurements using radiolabeled glycerol showed rapid glycerol uptake and turnover in saltern brines [20]. Many members of the class *Halobacteria* grow well on glycerol [4,21], but the species description of *Haloquadratum* *walsbyi*, the morphologically dominant type of *Archaea* in the brines studied, did not confirm efficient use of glycerol [22], and microautoradiography studies combined with fluorescence *in situ* hybridization did not show significant incorporation of glycerol into the flat square *Archaea* that dominated the prokaryote community in Spanish crystallizer ponds [23]. However, a gene annotated as *glpK*—glycerol kinase was found in the *Hqr. walsbyi* genome [24], and metagenomics studies of *Haloquadratum* in these brines showed all genes necessary for the efficient use of glycerol as growth substrate to be present [25]. Glycerol probably enters *Haloquadratum* cells by diffusion, as no specific uptake systems could be identified in its genome [24]. Glycerol can also be used as a growth substrate by *Salinibacter ruber* [26], in spite of the fact that it was not mentioned as a growth-stimulating compound in the species description [5], and a microautoradiography study of Spanish saltern brines did not show labeling of *Salinibacter* cells following incubation with radioactive glycerol [23].

Indications that glycerol may stimulate community respiration by the heterotrophic microorganisms in the Eilat crystallizer ponds, as well as in the Dead Sea, were already obtained in the mid-1990 in studies that monitored reduction of 2-(*p*-iodophenyl)-3-(*p*-nitrophenyl)-5-phenyl tetrazolium chloride (INT) to INT-formazan. Tetrazolium compounds compete with molecular oxygen for electrons in the terminal step of respiration. In the saltern pond samples glycerol caused a 52% increase in the rate of formation of formazan, which is similar to the degree of stimulation found in our oxygen uptake studies (Figure 2A). Stimulation of INT reduction by the Dead Sea heterotrophic community following addition of glycerol had a half-saturation constant of 0.75 μM. Similar to what was observed in our recent oxygen uptake studies, acetate and succinate did not increase the apparent respiration rate as assessed by INT reduction, while pyruvate was slightly stimulatory [27].

### 4.2. Dihydroxyacetone

Dihydroxyacetone was included in the experiments as it is excreted by *Salinibacter* as a partial oxidation product during metabolism of glycerol [26,28]. The identification of an efficient uptake system for dihydroxyacetone in the genome of *Hqr. walsbyi* [24] was the first indication that this compound may play a role in the ecophysiology of halophilic *Archaea* in the salterns. Similar systems for transport and metabolism for dihydroxyacetone were found in the genomes of isolates of *Spiribacter*, a genus of moderately halophilic representatives of the *Gammaproteobacteria* [29], recently identified as an important contributor to the prokaryote community in saltern ponds of intermediate salinity in Spain [30]. Dihydroxyacetone is efficiently used by *Haloquadratum* cultures and by the biota of the Eilat saltern crystallizer ponds [28]. The observed stimulation of the community respiration in the Eilat brines by dihydroxyacetone (Figure 2B) may therefore reflect the possible importance of this substrate in hypersaline environments.

### 4.3. Pyruvate

Another substrate found to stimulate respiration by the saltern crystallizer pond microbial community is pyruvate. Pyruvate is a favorite growth substrate for many species of extremely halophilic *Archaea*, including *Haloquadratum* [22]. There are additional species of the class *Halobacteria* that require pyruvate for growth, such as *Halosimplex carlsbadense*, an isolate from Permian rock salt collected from a salt mine in New Mexico, USA [31]. The metabolism of pyruvate by different halophilic microorganisms has recently been reviewed [32].

Pyruvate can be formed in hypersaline environments as a by-product of the metabolism of glycerol by certain halophilic *Archaea*. Species such as *Halorubrum saccharovorum*, *Haloarcula marismortui* and *Haloarcula vallismortis* form pyruvate and other acids (acetate, d-lactate) when supplemented with glycerol. This form of incomplete oxidation was observed to occur also in an archaeal bloom in the Dead Sea: labeled pyruvate, d-lactate and acetate could be detected even after addition of micromolar concentrations of ^14^C-glycerol [21]. The lactate was rapidly metabolized further after depletion of the glycerol, but acetate remained present for a prolonged time (see below).

### 4.4. Acetate

Experiments to assess the rate of uptake and turnover of acetate in the saltern crystallizer ponds of Eilat showed that its metabolism is very slow and that the microbial community has a low affinity for acetate [33]. Therefore, it is not surprising that emendation of crystallizer brine with acetate did not lead to a stimulation of the community respiration; on the contrary, a slight inhibition was sometimes observed (Figure 2C).

### 4.5. Fumarate

Experiments in which fumarate was added to Eilat saltern brines showed a significant decrease in oxygen uptake [16] (Figure 2D). This effect was explained by the observation that some halophilic *Archaea* can use fumarate as a terminal electron acceptor in respiration. Fumarate-driven anaerobic growth was reported in some strains of *Halobacterium salinarum*, in *Haloferax volcanii*, and in *Haloferax denitrificans* [34]. If indeed fumarate can relieve the need for molecular oxygen, especially when oxygen is already in short supply in salt-saturated solutions, fumarate respiration may be advantageous. However, the ecological relevance of fumarate as an electron acceptor in respiration remains to be ascertained as there is no indication that fumarate may be available in such hypersaline ecosystems. *Haloquadratum*, the dominant archaeon in the brines, probably cannot respire fumarate: no anaerobic growth in the presence of fumarate was observed [22], and no fumarate dehydrogenase genes were annotated in its genome [24]. Similarly, there is no information about the use or potential use of fumarate as an electron acceptor by *Salinibacter* [5,35].

## 5. Use of Oxygen Electrodes to Assess Primary Productivity in Saltern Crystallizer Ponds

Only one species of oxygenic phototrophs is generally found in saltern crystallizer ponds: the unicellular green alga *Dunaliella salina*, often colored orange due to its high content of β-carotene. However, we know surprisingly little about its photosynthetic activity *in situ*. Estimates of photosynthesis rates using conventional methods based either on ^14^CO_2_ incorporation measurements (e.g., [36]) or on oxygen evolution using the Winkler titration in ‘light’ and ‘dark’ bottles such as used in an older study of the activities along the salinity gradient in a Spanish saltern system [15] are all problematic because of methodological constraints due to the special nature of the brines and the organisms involved. These issues were summarized in earlier review papers [1,37]. In the latter experiment, primary production in the crystallizer ponds (>300 g/L salt) was below the detection limit, in spite of the massive presence of *Dunaliella* (3.5 μg/L chlorophyll *a*) [15].

Attempts have been made to monitor diel oxygen changes in mesocosms by measuring changes in oxygen concentrations, using chemical assays (the Winkler titration) or by electrodes. Production estimates of ~0.8–1.5 µmol O_2_/L·h, were obtained for brines with 1300–2100 *Dunaliella* cells/mL [19]. A light-dark shift experiment in which Eilat crystallizer brine (1100 *D. salina* cells/mL) was incubated at 35 °C in the above-described 630-mL incubation chambers provided with oxygen optodes yielded calculated production rates of ~1 µmol O_2_/L·h, equivalent to ~9 × 10^−13^ mol O_2_/*Dunaliella* cell·h (unpublished results). However, the finding that light-dark shifts may also influence the respiration rate of the archaeal community in the salterns due to the activity of bacteriorhodopsin and similar light-driven proton pumps (see below) implies that it not possible to calculate rates of oxygenic photosynthesis using the experimental setup employed.

## 6. The Possible Effect of Bacteriorhodopsin and Other Light-Driven Proton Pumps on the Community Respiration in Saltern Crystallizer Ponds

Many extremely halophilic *Archaea*, including *Haloquadratum* which often dominates the community of saltern crystallizer ponds, contain bacteriorhodopsin, a retinal protein that functions as a light-driven proton pump and enables direct use of light energy to generate a proton gradient over the membrane that can be converted to ATP [2,4]. Certain light-driven proton pumps in different taxa belonging to the class *Halobacteria* are known under other names such as archaerhodopsin and cruxrhodopsin. *Salinibacter* possesses a similar membrane-bound light-driven proton pump named xanthorhodopsin [38]. In the section below, all such proton-pumping retinal proteins are designated as ‘bacteriorhodopsin’.

It has been known since the early 1970s that illumination of bacteriorhodopsin-containing cultures of *Halobacterium* results in a decrease in respiration rate. This effect can be explained based on competition between two energy yielding systems: (1) the respiratory chain that produces a proton gradient based on electron flow from the oxidation of organic substrates and (2) conversion of light energy to a proton gradient by bacteriorhodopsin [39]. The effect was later exploited to assess the action spectrum of light utilization in *Salinibacter* by the xanthorhodopsin-salinixanthin pigment system [38,40]. We therefore asked the question whether a similar light-dependence may exist for the community respiration by the heterotrophic microorganisms in saltern crystallizer ponds.

To assess the presence of bacteriorhodopsin and similar retinal proteins in the crystallizer brine of Eilat we recorded absorption spectra of biomass collected by centrifugation against samples in which the retinal pigments had been bleached by cetyltrimethylammonium bromide. The bacteriorhodopsin content of the brine (3.5 × 10^7^ prokaryotes/mL) was estimated at ~3.6 nmol/L [18]. Normalized for the community density this value is very similar to the value of 0.4–0.6 nmol/L for 5 × 10^6^ prokaryote cells/mL in the Dead Sea in 1981 [41] and 2.2 nmol/L reported for brines with ~10^7^ prokaryotes/mL from the Exportadora de Sal, Baja California [3], based on a different assay method. The difference spectra of non-bleached *versus* bleached biomass samples from Eilat showed a peak around 583 nm [18], similar to the wavelength reported (~580 nm) for biomass collected from the Dead Sea during an archaeal bloom in 1980–1981 [41]. *Haloquadratum walsbyi* cultures treated in a similar way gave a peak at 579 nm [18]. These peaks, measured in crude membrane preparations, are red-shifted compared to the value of 540 nm reported for purified ‘Squarebop I’ bacteriorhodopsin from the salterns at Margherita di Savoia, Italy, or the 551 and 540 nm peaks found in dark-adapted *Hqr. walsbyi* strain HBSQ001 [42,43].

In experiments intended to assess the effect of light on the heterotrophic respiration of the community, photoautotrophic oxygen production by *Dunaliella* and any other phototrophs that may be present must be abolished. Therefore, the experiments were performed in the presence of 5 μM 3-(3-4-dichlorophenyl)-1,1-dimethylurea (DCMU), an inhibitor of photosynthetic electron transport at the acceptor side of photosystem II. Control experiments showed that DCMU at this concentration fully inhibits photosynthetic oxygen production by *D. salina* in the brine. The ethanol used to dissolve the inhibitor did not greatly affect dark respiration rates at the concentration added (17 mM), which is consistent with the fact that ethanol is not known as a preferred substrate for the growth of members of the *Halobacteria* [18].

Upon illumination the oxygen consumption rate of an Eilat crystallizer brine sample supplemented with 5 μM DCMU decreased by 40%–43% (Figure 3). The simplest explanation for this phenomenon is the competition between two energy yielding systems. When light is available and the bacteriorhodopsin proton pump can operate, the halophilic *Archaea* and other organisms that possess retinal-based proton pumps need to waste less available organic substrates, which anyhow may be in short supply, to generate energy by aerobic respiration [18]. The extent of the decrease in respiration inhibition in the light was even larger than the ~30% decrease in the rate reported for bacteriorhodopsin-containing cultures of *Hbt. salinarum* [39]. Our results thus suggest that photons may supply a significant part of the daytime energy demand of the halophilic prokaryotes present in the community.

## 7. Final Comments

The above-presented data show that simple experiments using oxygen sensors can teach us much about the *in situ* activities and the potential activities of the different components of the biota inhabiting solar saltern crystallizer ponds: the oxygenic photoautotrophic alga *Dunaliella*, and *Archaea* of the class *Halobacteria*, often accompanied by minor communities of *Salinibacter* and possibly other members of the domain *Bacteria*.

Changes in oxygen concentration in hypersaline solutions can be assessed in different ways. Different modifications of the Winkler titration have been occasionally used for the purpose [12,15,16], but each sample can be analyzed only once, and therefore continuous monitoring of changes in the oxygen content is not possible. Clark-type oxygen electrodes generally function poorly at high salt. Moreover, oxygen is consumed at the cathode, so that the measuring system may influence the availability of oxygen in the system, which is anyhow low due to its limited solubility at high salt concentrations. However, needle microelectrodes have been successfully employed in the study of hypersaline ecosystems [44]. The limited experience gained thus far with applications of optical oxygen sensors in hypersaline brines [16,17,18] shows that such sensors can perform well, are stable, and can be calibrated at the *in situ* salinity.

Many, or possibly even most extremely halophilic *Archaea* and *Bacteria* not only grow as aerobic chemoheterotrophs, but also have a considerable potential of photoheterotrophic growth mediated by light absorption by bacteriorhodopsin and similar light-driven proton pumps. It was shown that the oxygen consumption by the prokaryote component of the community was significantly lower in the light than in the dark. This effect could be attributed to the use of photons as energy source to replace part of the energy to be obtained by aerobic respiration of organic substrates.

Earlier models describing the functioning of hypersaline ecosystems such as saltern crystallizer brines often failed to take the light-driven proton pumps into account as a major factor in the energy generation of the system. In addition, it was always tacitly assumed that oxygen production in the light and oxygen consumption in the dark can be used to estimate oxygenic photosynthesis and aerobic respiration [19]. The fact that light excitation of retinal-based proton pumps may cause a significant decrease in respiration of the (photo)heterotrophs that dominate the prokaryotic community in the system now requires a critical re-evaluation of all older data on primary productivity in salt lakes and saltern ponds [1,15,37,45], as respiration of the prokaryotic heterotrophic component of the community may be strongly light-dependent.

All this does not imply that assessment of primary productivity in saltern crystallizer ponds or in natural salt lakes with comparable salinity can better be performed based on measurements of radiolabeled CO_2_ incorporation in the light and in the dark [1,36,37]. Such methods are also marred by problems due the fragility of *Dunaliella* cells that easily break during filtration. Moreover, there even are reports showing that light absorption by the bacteriorhodopsin proton pump may under certain conditions affect CO_2_ incorporation by halophilic *Archaea* as well, so that light-dependent CO_2_ fixation is not necessarily uniquely due to oxygenic photosynthesis [46,47]. In summary, there is currently no simple and straightforward approach to reliably estimate the photosynthetic activity by *Dunaliella* or the respiratory activity of the prokaryotic communities during diel light-dark cycles in salt-saturated saltern ponds and in natural hypersaline lakes that support the development of similar microbial communities.
